# Supplementary material for: SyNDI: synchronous network data integration framework
Source: BMC Bioinformatics. 2018 Nov 6;19:403. doi: 10.1186/s12859-018-2426-5 (PMC6219086; doi:10.1186/s12859-018-2426-5)
Supplement: Supplementary file 5 — Files of the example presented in Additional file 4. This additional file is a zip package that contains all files used in Additional file 4. Detailed descriptions of each of these files are presented in a README file included in this zip package. (GZ 30411 kb) [file 12859_2018_2426_MOESM5_ESM.gz › SyNDITools/Meme2Fimo/meme_4.9.0_4.tar.gz!/meme_4.9.0/website/html/glam2scan-intro.html]

GLAM2 - Overview


|  |  |
| --- | --- |
|  | MEME Suite The MEME Suite web application requires the use of JavaScript   Javascript doesn't seem to be available on your browser.  Overview  GLAM2SCAN is a tool for finding occurrences of a GLAM2 motif in a sequence database.  Because GLAM2 motifs may contain gaps, standard search tools such as MAST and FIMO cannot handle them. Hence the need for GLAM2SCAN.  Here is a GLAM2SCAN sample output. GLAM2SCAN reports a score for each site, higher scores indicating better matches to the motif. The output includes the top 25 highest-scoring sites. |
